# Retraction: Japanese encephalitis virus orchestrates GLUT4-mediated glucose metabolism to potentiate viral replication via insulin receptor signaling

**DOI:** 10.1371/journal.ppat.1014600

**Published:** 2026-09-21

**Authors:** The PLOS Pathogens Editors

After this article [1] was published, concerns were raised regarding results presented in Figs 2, 4, 6-8, S2, and S4.

Specifically:

- The following lanes appear more similar than expected from independent results:
  - Fig 2L β-actin lanes 4 and 5
  - Fig 4A GLUT1 lanes 1 and 2
  - Fig 4J GLUT3 A549 lanes 3 and 4
  - Fig 8F p-AS160 lanes 9 and 10
  - Fig S2A GLUT3 lanes 2 and 4
  - Fig S4B p-PI3K lanes 2 and 5
- There appear to be repetitive features in the background noise within the Fig 6H IP:HA panel, and between the Fig 6H IP:HA and Fig 7K IP:IRS1 HA panels.
- The Fig 6C GLUT4 SC79 panel appears similar to Fig 8C GLUT4 panel lanes 2–5.

Corresponding author BZ stated that the Fig 6C GLUT4 SC79 panel is incorrect due to a figure preparation error, and they provided a replacement panel and underlying uncropped blot data.

Regarding the Figs 6H and 7K similarities, corresponding author BZ stated that a local background processing step had been used during assembly of the Fig 6H IP:HA panel to remove minor background imperfections.

Corresponding author BZ provided underlying data for the remainder of the above concerns about Figs 2L, 4A, 4J, 8F, S2A, and S4B. Upon editorial review, the comments and data provided did not resolve all of these concerns.

In light of the above concerns that question the reliability of the reported results, the PLOS Pathogens Editors retract this article.

YYG agreed with the retraction and apologizes for the issues with the published article. BZ did not agree with the retraction and stands by the article’s findings. QW, PH, SYZ, JFZ, SF, QD, JCW, and JC either did not respond directly or could not be reached.
